# Supplementary material for: Causal Relationship between Aging and Anorexia Nervosa: A White-Matter-Microstructure-Mediated Mendelian Randomization Analysis
Source: Biomedicines. 2024 Aug 16;12(8):1874. doi: 10.3390/biomedicines12081874 (PMC11351342; doi:10.3390/biomedicines12081874)
Supplement: Supplementary file 1 [file biomedicines-12-01874-s001.zip › Supplementary Figure/Figure S9/Leaveoneout plot of MR analysis for ubm-b-1834 to AN.pdf]

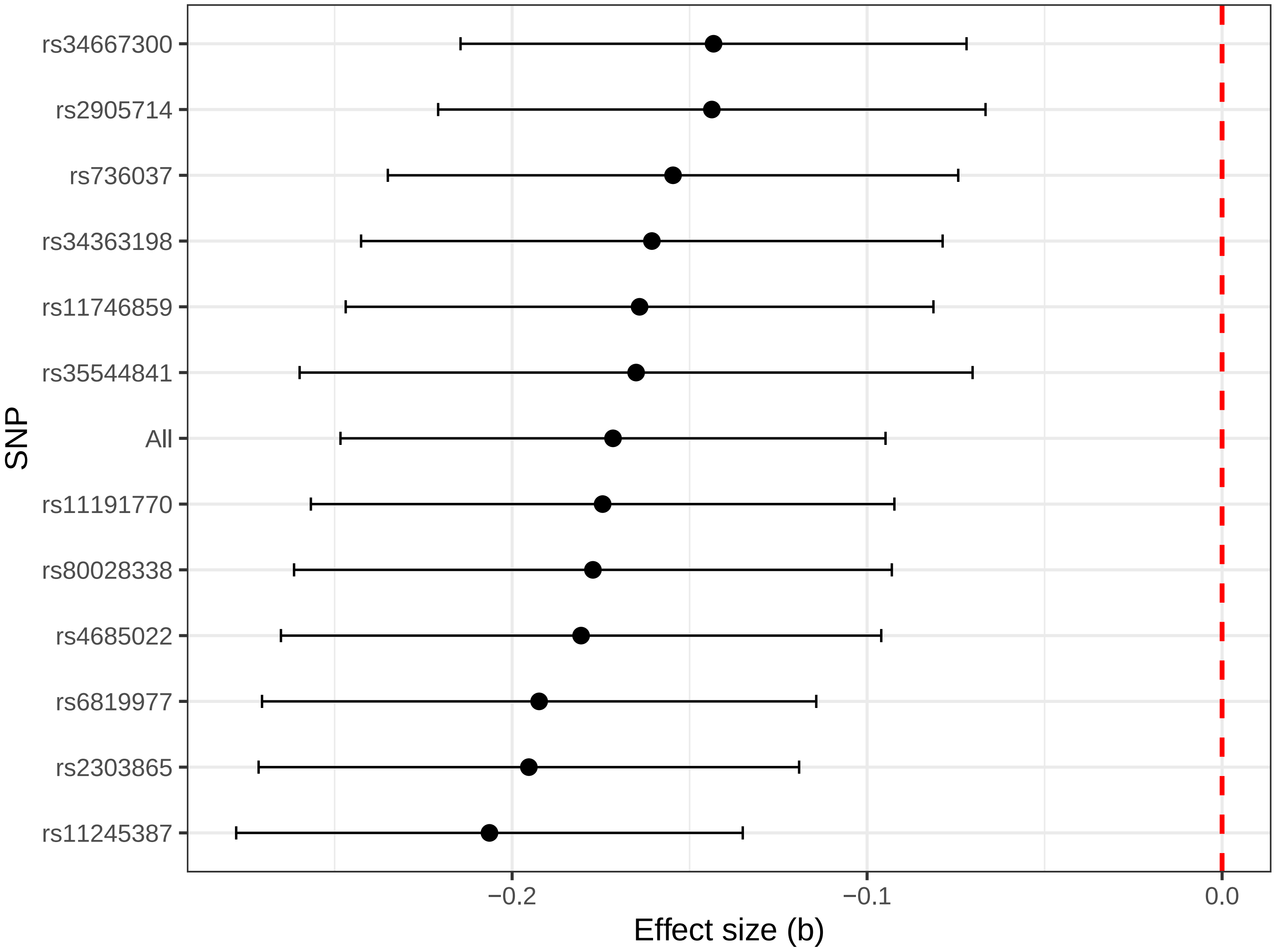

rs34667300
rs2905714
rs736037
rs34363198
rs11746859
rs35544841
All
rs11191770
rs80028338
rs4685022
rs6819977
rs2303865
rs11245387
SNP
−0.2
−0.1
0.0
Effect size (b)
